# Invasive Breast Cancer Incidence in 2,305,427 Screened Asymptomatic Women: Estimated Long Term Outcomes during Menopause Using a Systematic Review

**DOI:** 10.1371/journal.pone.0128895

**Published:** 2015-06-24

**Authors:** Winnifred Cutler, Regula Bürki, James Kolter, Catherine Chambliss, Erika Friedmann, Kari Hart

**Affiliations:** 1 Department of Women’s Health Research, Athena Institute for Women’s Wellness, Chester Springs, Pennsylvania, United States of America; 2 Department of Gynecology, Hirslanden Hospital Group, Bern, Switzerland; 3 Department of Obstetrics and Gynecology, The Philadelphia College of Osteopathic Medicine, Philadelphia, Pennsylvania, United States of America; 4 Department of Obstetrics and Gynecology, Paoli Hospital, Paoli, Pennsylvania, United States of America; 5 Department of Psychology, Ursinus College, Collegeville, Pennsylvania, United States of America; 6 School of Nursing, University of Maryland, Baltimore, Maryland, United States of America; 7 Department of Mathematics and Computer, The Hotchkiss School, Lakeville, Connecticut, United States of America; Kyushu University Faculty of Medical Science, JAPAN

## Abstract

**Background:**

Earlier studies of breast cancer, screening mammography, and mortality reduction may have inflated lifetime and long-term risk estimates for invasive breast cancer due to limitations in their data collection methods and interpretation.

**Objective:**

To estimate the percentage of asymptomatic peri/postmenopausal women who will be diagnosed with a first invasive breast cancer over their next 25 years of life.

**Methods:**

A systematic review identified peer-reviewed published studies that: 1) enrolled no study participants with a history of invasive breast cancer; 2) specified the number of women enrolled; 3) reported the number of women diagnosed with a first invasive breast cancer; 4) did not overcount [count a woman multiple times]; and, 5) defined the length of follow-up. Data sources included PubMed, Cochrane Library, and an annotated library of 4,409 full-text menopause-related papers collected and reviewed by the first author from 1974 through 2008. Linear regression predicted incidence of first invasive breast cancer, based on follow-up duration in all studies that met the our inclusion criteria, and in a subset of these studies that included only women who were 1) at least 50 years old and 2) either at least 50 or less than 50 but surgically menopausal at enrollment.

**Results:**

Nineteen studies met the inclusion criteria. They included a total of 2,305,427 peri/postmenopasual women. The mean cumulative incidence rate of first invasive breast cancer increased by 0.20% for each year of age (95% CI: 0.17, 0.23; p < 0.01; R^2^ = 0.90). Over 25 years of follow-up, an estimated 94.55% of women will remain breast cancer-free (95% CI: 93.97, 95.13). In the 12 studies (n = 1,711,178) that enrolled only postmenopausal women, an estimated 0.23% of women will be diagnosed with a first invasive breast cancer each year (95% CI: 0.18, 0.28; p < 0.01, R^2^ = 0.88).

**Conclusion:**

The vast majority (99.75%) of screened asymptomatic peri/postmenopasual women will not be diagnosed with invasive breast cancer each year. Approximately 95% will not be diagnosed with invasive breast cancer during 25 years of follow-up. Women who receive clinical examinations, but do not have mammograms, will have higher cancer-free rates because innocuous positives (comprising 30-50% of mammography diagnoses) will remain undetected. Informed consent to asymptomatic women should include these results and consideration of the benefits of avoiding mammograms.

## Introduction

The value of routine breast cancer screening for women, and the dangers of breast cancer overdiagnosis [1] lately have been matters of heated debate. Recent studies compellingly demonstrate that routine mammography screening frequently results in overdiagnosis because it identifies invasive breast cancers that would either have regressed on their own, or never developed to clinical significance. Overdiagnosis may account for 30%-50% of cancers identified by mammography screening [2–4]. The absolute number of overdiagnoses may exceed 70,000 women in the U.S. each year [4].

The overdiagnosis of breast cancer increases women’s stress and fear, and also results in unnecessary and invasive surgical and medical treatments. Treatments can be painful, lead to adverse events, create financial burden, inhibit sexual relationships, and increase the risk of other diseases, including ischemic heart disease [5–7].

Overdiagnosis also distorts mortality rate calculations because the mortality rate is defined as the number of women who died divided by the number diagnosed. Increasing the number of diagnosed women by 30–50% in the screened group lowers the apparent mortality rate [8]. The belief that early diagnosis has reduced breast cancer mortality in the populations of screened women is caused largely by a failure to appreciate the impact of overdiagnosis, which creates an artificial drop in the mortality rate.

Breast cancer incidence figures were the subject of a major 2009 report sponsored by the U.S. Centers for Disease Control (CDC). It covered 92.1% of the US population over a six-year period (1999–2004) and identified 905,402 invasive and 233,833 in situ breast cancer cases from the National Program of Cancer Registries [9]. The CDC authors calculated age-grouped annual rates of breast cancer based on numbers of cases and census estimates. Women older than 39 accounted for 94.7% of invasive breast cancer cases and 96.6% of in situ cases. Women over 69 accounted for 32.2% of invasive and 24.9% of in situ cases. These CDC data revealed that 20% of all registered breast cancer cases were in situ [9]. United Kingdom (UK) data showed a similar in situ rate of 17.6% of all breast cancers [10]. While in situ cancers are not lethal, they do trigger increased surveillance for early invasive breast cancer.

In 2013, the CDC posted on its consumer information website that “women have a 13% lifetime risk of developing breast cancer” [11]. The CDC reports may inflate lifetime risk because of three closely related limitations in their data collection and interpretation techniques. First, the CDC data report the number of breast cancer cases [9, 12] rather than the number of women who have breast cancer; this means some women are counted more than once. The CDC explains their method of calculating incidence in this way: “numerator of the incidence rate is the number of new cancers… and may include multiple primary cancers occurring in one patient” [12]. For example, if a new tumor is found in a new location, or if the histology of a recurrent cancer differs from that of the primary cancer, the same woman will be counted more than once. Women who move to a different region and attend a new clinic may also be incorrectly counted as new cases. Unfortunately, the CDC provided no data on tabulating individual women, and did not report the number of duplications [9].

Second, women who have had breast cancer are much more likely to be diagnosed with a new primary breast cancer than women who have never had cancer [13]. Since the CDC considers each primary tumor, and recurrence with a different histology, a new case, the incidence rate is inevitably inflated as the population ages because as women age it is increasingly likely that screening will reveal a second, third or fourth tumor. When the CDC overcounts tumors in older women it makes the likelihood of a first breast cancer appear to increase as they age. But this apparently higher incidence of cancer in older women may, at least in part, be caused by counting the same women repeatedly.

Third, census data estimates suffer from the now-documented problem of undercounted populations [14–17], which may affect breast cancer incidence estimates that are based on such census data [16,17]. For example, census survey respondents may undercount household members; this inflates cancer incidence rates. Although census data are collected in a single year, they are then estimated for the subsequent nine years, and the problem of undercounted household members compounds over time. For example, in Marin County, California, the 2000 census-estimators made an error, underestimating the number of household members by 32%. This increased the calculated breast cancer rate among women 50–69 years by an astonishing 50% [15]. Although these undercounts and census-related estimation problems have been acknowledged, they continue [16,17].

These limitations affect both the numerator of the incidence fraction that is increased as women are overcounted, and the denominator that is reduced when the population is undercounted. The effects may compound when long-term risk estimates are calculated from these numbers. The CDC consumer website states that a woman has a 13% lifetime risk of being diagnosed with breast cancer, generating widespread misperception that this applies to all groups of women. If estimates of both short and long-term breast cancer risk are not accurate, the decisions asymptomatic women and their doctors make about screening cannot be well informed. We seek to provide more reliable estimates across time by stage of life by using accessible published data that overcome these artifacts of overcounting numbers of women and undercounting estimated sample size.

The goal of our study was to estimate the percentage of asymptomatic women with no cancer history who will be diagnosed with a first invasive breast cancer in their peri/postmenopausal years, and to determine the likelihood that these women will remain breast cancer-free. This systematic review included published incidence data from cohort studies, screening studies, and randomized trials that examined incidence of new invasive breast cancer. It included studies that both pre-cleared women for breast cancer history and tracked potential emergence of breast cancer diagnosis. Studies focusing on other outcomes were included if they met these criteria. For example, the Women's Health Initiative randomized controlled trials, which were designed to test cardiovascular health benefits of hormonal therapy, also provided a rich source of breast-screening information.

Studies were excluded if they counted a woman multiple times or relied solely on population census estimates to predict the likelihood those women would be diagnosed with breast cancer. After the rigorous selection, linear regression analysis was used to model the cumulative incidence of a first invasive breast cancer based on the duration of follow-up among enrolled women.

## Methods

A multi-step, systematic search protocol identified all peer-reviewed published studies in which participants had been screened for a first invasive breast cancer and met five inclusion criteria: 1) Study participants were peri/postmenopausal women with no invasive breast cancer history at enrollment; 2) The study specified the number of women enrolled; 3) The study specified the number of women diagnosed with a first invasive breast cancer; 4) Study subjects were counted only once; and, 5) The duration of follow-up was clearly defined. Studies were excluded if they only estimated the number of women screened, followed, or diagnosed with breast cancer.

We examined a unique index of published papers in the field of reproductive and behavioral endocrinology of aging women compiled by the first author, Winnifred Cutler (WC) from 1974–2008 for 7 textbooks she wrote (S2 Table). The index consisted of menopause-related papers that had been reviewed, outlined and indexed. Importantly, the index included methodology and findings about breast cancer diagnoses in papers primarily focused on other topics. The cross-indexing system enabled identification of the papers pertinent to the current study. Outlines of these studies were reviewed, to evaluate whether the study met inclusion criteria. When necessary, the full-text publications were retrieved for closer examination. (S1 Text; S1 File; S2 File; S2 Text).

Article identification continued (Step 1) with a PubMed search for references using the search terms "breast cancer and menopause or hormone therapy (or HRT)" for articles published from 2008 through June 2012 (S2 Table). Dr. Burki and Dr. Kolter scanned their medical society notices also contributing any references identified by their routine medical education reports over the 2-year active phase of article identification. These results were combined with the outlines extracted from WC’s database.

At least one coauthor and two independent readers (Debbie McLaughlin [DM], Emily Short [ES]) reviewed every study that reported findings on breast cancer incidence. WC assessed eligibility, based on the five inclusion criteria. DM and ES also reviewed the selections. Disagreements were resolved by consensus.

Two searches of the Cochrane Library databases (Steps 2 and 3) subsequently identified all eligible screening trial articles. For the first search we used the phrase, “Breast cancer incidence during screening,” (S2 Table), and for the second search we used the phrase, “Mammography or hormone use and breast cancer” (S2 Table). Both searches were current to September 28, 2012. Finally, citations in the papers that had been identified were examined to discover additional articles to evaluate; these did not return additional studies.

No language or study design restrictions were imposed on any of the searches. When multiple publications referenced the same study, we included the latest publication that reported on the complete enrolled sample of women. Three readers (WC, DM, and ES) reviewed potentially eligible studies and excluded those that did not meet the criteria.

After determining potential eligibility, the three readers extracted information from eligible studies: number of women enrolled; number of women diagnosed with a first invasive breast cancer; length of follow-up; and, characteristics of trial participants and inclusion/exclusion criteria (including age at enrollment, cancer history, surgical history, health habits, and environmental exposures). For six studies, we contacted the original investigators to verify that their study met our inclusion criteria and the counts we extracted were accurate.

The primary outcome of interest for review and subsequent data analysis was the total cumulative incidence of invasive breast cancer for population subgroups. To minimize potential sources of non-response and selective outcome bias, we included only studies where outcomes were reported for all enrolled women who were followed up for a similar, specific length of time.

### Description of Statistical Analyses

A least squares linear regression was used to model the cumulative incidence of a first invasive breast cancer, based on the duration of follow-up among enrolled women. We used this model to generate a summary estimate of the cumulative incidence rate of a first invasive breast cancer for a given follow-up period, with corresponding 95% confidence limits. The model allowed us to quantify the effect of longer follow-up duration on the cumulative total incidence of invasive breast cancer.

#### Model Diagnostics to Assess Validity of Linear Regression

The 5 assumptions required for a least squares linear regression model are 1) existence, 2) independence, 3) homogeneity of variance, 4) linearity, and 5) normality. ***Existence*** is met since, for any fixed follow-up duration, the cumulative incidence of invasive breast cancer will have a probability distribution with finite mean and variance. ***Independence*** requires that the cumulative incidence of first invasive breast cancer be independent for each study and was assessed via careful consideration of the women and studies included in the systematic review. ***Linearity*** requires that the relationship between follow-up duration and average cumulative incidence of first invasive breast cancer be linear. ***Homogeneity of variance*** requires that the variance in the cumulative incidence of first invasive breast cancer is the same for any duration of follow-up. We assessed linearity and homogeneity of variance by inspecting a Scatterplot that related cumulative incidence of first invasive breast cancer to duration of follow-up and corresponding residual plots. ***Normality*** requires that, for any fixed duration of follow-up, the cumulative incidence of first invasive breast cancer is normally distributed; this was verified by a normal probability plot of the residuals.

Since all assumptions for least squares linear regression were met, this statistical approach gave us an advantage over meta-analysis since it did not require pooling information from heterogeneous studies. For detecting invasive breast cancer in women it is useful to avoid pooling, because among asymptomatic women, for every five-year age group between 45–70, a screened group’s incidence of breast cancer identified is much higher in a first mammogram screening than in subsequent rescreening examinations (see S3 Text) [10].

Hence, linear regression offers a reasonable estimate of long-term freedom from a diagnosis of invasive breast cancer. It uses only readily available, published information, rather than requiring information that is impossible to obtain, such as number of rescreenings.

After we conducted regression analysis for all studies, we conducted parallel analyses for two subgroups by fitting separate linear regression models to studies in which women were 1) at least 50 years old at enrollment, and 2) at least 50 years old at enrollment or younger than 50 and surgically menopausal.

#### Undue Influence Bias

To minimize bias and ensure that no single study had an undue influence on the results of the regression analysis, we closely examined studies that reported substantially higher/lower incidence rates than the regression model predicted. A sensitivity analysis was performed by a parallel regression analysis after we eliminated the one study that followed women for 25 years (11 years longer than any other study).

## Results

**Fig 1:**
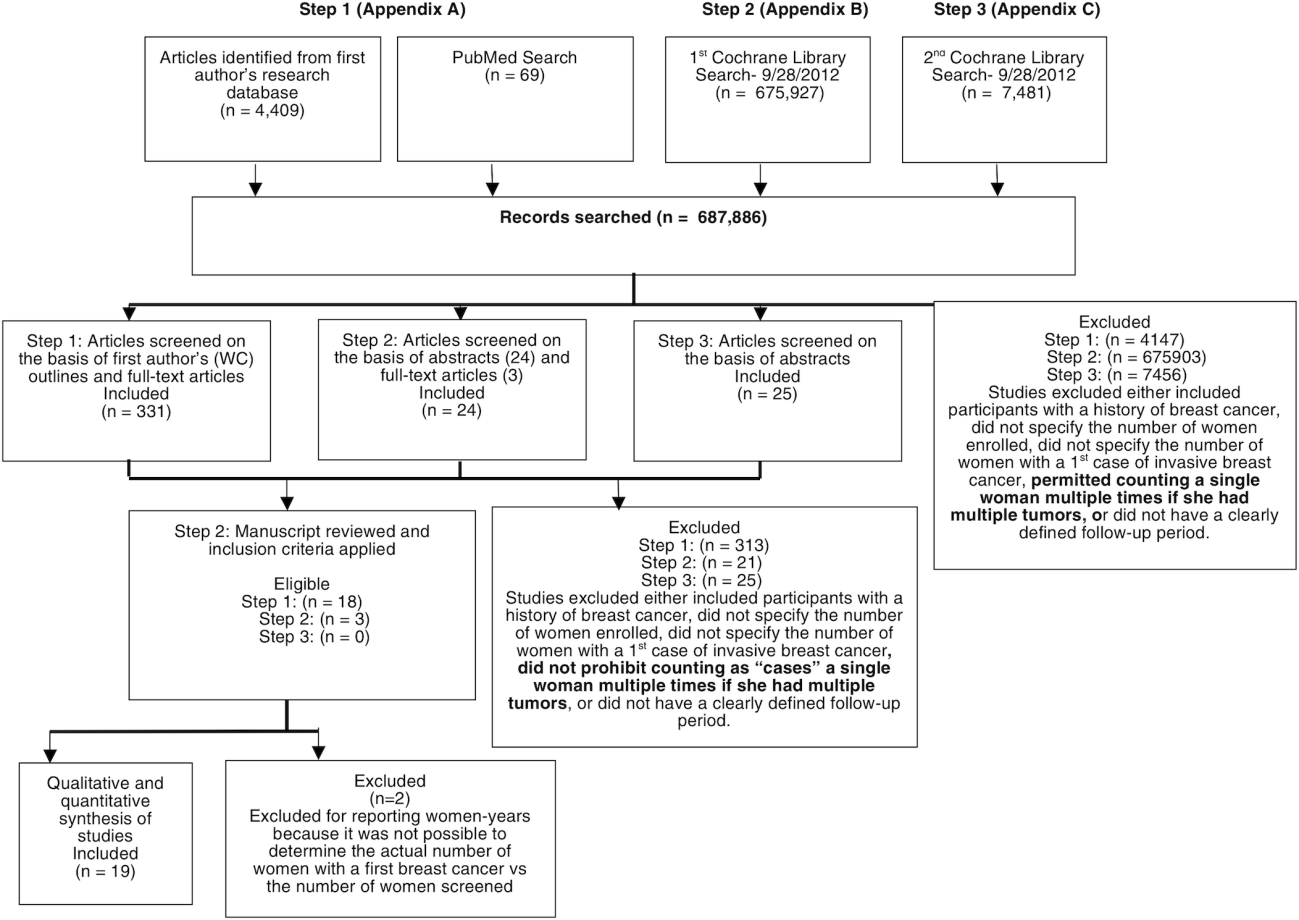
The Flowchart of Search Steps for Retrieval of 19 Qualified Studies.

A total of 687,886 records of peer-reviewed published articles were searched for relevant studies. WC’s research database contained 4,409 potentially relevant articles, and the PubMed Search, combined with those of Drs. RB and JK, returned 69 more (Step 1). Of these, we screened 331 full-text articles and identified 18 potentially eligible candidates. (S2 Table) Our first search of the Cochrane Library database (Step 2) scanned 675,927 articles, returned 24, and identified three potentially eligible candidates (S2 Table). The second search of the Cochrane Library database (Step 3) scanned 7,481 articles, and returned 25 records; none were eligible (S2 Table). Two studies reported in woman-years, which made it impossible to determine how many of those screened had a first breast cancer, and these studies were excluded. Nineteen studies met the inclusion criteria and were included in the review and subsequent analyses (see Fig 1).

Table 1 shows the design of each of the original studies and notes all interventions.

**Table 1:** Research design details of 19 published studies of 2,305,427 peri/postmenopausal women screened for breast cancer.

	Trial	DESIGN DETAILS
1	UK Million Women [18]	Population based **cohort study**: postmenopausal women, 25% with hysterectomy, and 11% with bilateral ovariectomy. All without a prior cancer recruited 1999–2001 to evaluate HRT impact in a breast cancer-screening program with mammograms. 1·4% excluded because of a prior diagnosis of cancer. **1st incident of invasive breast cancers.**
2	Danish Nurses [19]	Prospective **cohort study** testing HRT relationship to breast cancer identified by linkage via unique ID# to the Danish nationwide registries. Women with prior breast cancer excluded. Follow-up for incident cases started with the questionnaire in 1993, ended 1999. **1st incident of invasive breast cancers.**
3	Melbourne [20]	Collaborative **cohort study**: History of HRT use among women postmenopausal at baseline within 5 years before enrollment. No woman had any cancer in history [other than in situ lobular breast cancer] in the 5 years before enrollment. Southern European migrants over-sampled to increase demographic range. **1st incident of invasive breast cancer.**
4	Finnish Registry I [21]	**Cohort Study** All Finnish women >50 using estrogens ≥6 months were identified from the national medical reimbursement register and followed for breast cancer using the national cancer registry. Women w. prior br. ca excluded. Since 1987, 90% of Finnish women have taken part in mass mammogram screening programs that offer a free-of-charge mammogram every 2^nd^ year to all women between 50 and 60 with many also to age 65. **1** ^**st**^ **incident cases of invasive breast cancers**
5	Finnish Registry II [22]	**Cohort study** All Finnish women >50 using estrogens-progestogen ≥6 months were identified from the national medical reimbursement register and followed for breast cancer using the national cancer registry. Women with prior breast cancer excluded. Since 1987, 90% of Finnish women have participated in mass mammogram screening programs offering a free-of-charge mammogram every 2nd year to all women between 50 and 60 with many also to age 65. **1** ^**st**^ **incident cases of invasive breast cancers.**
6	French Cohort [23]	**Cohort study**: 3175 postmenopausal women, comparing breast cancer in HRT users vs. non-users among women without prior breast cancer. **1st incident of invasive breast cancer.**
*7*	WHI I [24]	**RCT** primary prevention trial. Women 50–79 with intact uterus and no prior breast cancer. Mammography screenings and clinical breast examinations at baseline and then annually. Invasive breast cancer in placebo vs. Prempro. All invasive breast cancers are counted, but number of patients is shown; so **incidence = 1st incidence**
8	WHI II [25]	**RCT** primary prevention trial. Women 50–79 without uterus and no prior breast cancer. Placebo *vs*. Premarin Mammography screenings and clinical breast examinations as WHI I. **1st incident of invasive breast cancer.**
9	SwedenGothenburg Screening Trial [26]	**RCT** randomized into screened and control groups of women without prior breast cancer. Started in 1982 to show that mammogram screenings reduced mortality. Women>49 had 4; the rest had 5 invitations to screening every 18 months. 7 years screening then follow-up data for breast cancer carefully tabulated from cancer registries >5 years. **1st incident of invasive breast cancer**
10	UK Trial of Early Detection of Breast Cancer (TEDBC) [10]	**Cohort study** comparing screened women to other groups enrolled at the same time. But study does not say if it excluded those with a prior breast cancer. Only the 2 cohorts of screened women are analyzed. **Incident invasive breast cancer, one case per person.**
11	Australia Record Review [27]	**Record review** of referral center for testosterone supplementation for HRT users. Women with prior breast cancer not included. All doses titrated individually. Baseline then biannual mammograms. **1st incident of invasive breast cancer.**
12	Osteoporosis Fracture Study [28]	**Prospective data** from community based women age 70+5 with no prior breast cancer. Baseline estradiol level did not predict subsequent breast cancer. **1st incident of invasive breast cancer.**
13	Italy ORDET [29]	Prospective **cohort study** of postmenopausal women with intact ovaries who contributed baseline blood samples to test whether baseline steroid levels were predictive of subsequent developing invasive breast cancer (histologically confirmed). None with prior cancer or liver disease. **1st incident of invasive breast cancer**
14	N Y U [30]	Prospective **cohort study** of New York City postmenopausal women, w. no prior breast cancer who received screening for breast cancer at the time of blood sampling. **1st incident of invasive breast cancer**.
15	US Breast Cancer Detection Demonstration Project [31]	Breast exams at **29 screening centers in 27 US cities** providing total number of women without prior breast ca, and number of women with breast cancer after screening, reported at the start of this follow up study of physical activity and risk. **1st incident of invasive breast cancer** 17% of the cases were *in situ not shown in table*. 89% Caucasian.
16	Sweden-Malmo [32]	25 year prospective **cohort study** of women with no prior breast cancer, showing 19% overdiagnosis in women 55–69 with mammogram screening: All enrolled 1976, followed until 2002, comparing approximately 50% with *vs*. 50% without regular mammograms every 12 to 18 months during initial screening phase. Total cumulative incidence of breast cancer reported. **1st incident of invasive breast cancer.**
17	Norwegian Cohorts [33]	**Cohort study.** Randomized screening (3 times) showing 22% overdiagnosis in women 55–69. Post randomization mammography offered to all women <70. Total cumulative incidence of invasive cancer reported. **1st incident of invasive breast cancer.**
18	Swedish Two County Trial [34]	**Randomized enrollment** of women without prior breast cancer to active and passive screening groups in 1977, with the screening group results shown. Designed to show long-term benefit of mammogram screening in reducing mortality from breast cancer. 85% accepted screening that continued for 7 consecutive years, 123 in situ cancers omitted. Author confirmed that only first cancer is recorded. **1st incident of invasive breast cancer.**
19	Canadian National Breast Screening Study [35]	Women without prior breast cancer, **randomized** into annual mammogram and clinical examination or just clinical examination, with thorough training of the nurses and doctors to conform to clinical examination protocol. During 8 years the number of invasive breast cancers found was equivalent as were mortality outcomes. This is the only systematic screening study to compare protocol-trained annual clinical breast examination alone to clinical exam plus mammogram screening. Mammograms do produce an earlier lead-time, more biopsies and surgeries, but no savings in mortality. Invasive breast cancers are reported: there were 388 prevalent cases within 6 months of enrollment and 944 more during the 8 years of continuous follow-up. **1st incident of invasive breast cancer.**

The 19 studies we included collectively document 2,305,427 peri/postmenopausal women from 10 countries (see Table 2). Of these women, 34,514 were diagnosed with a first invasive breast cancer through systematic screening and follow-up, which yielded a net invasive breast cancer incidence of 1.50% with mean follow-up of 8.1 years [10, 18–35].

**Table 2:** Percent screened Peri/Postmenopausal Women Diagnosed with 1^st^ Incident Invasive Breast Cancer in 19 published Studies.

#	Trial	Number of Women	Baseline Age	Years Studied	Number with Breast Cancer	Incidence [Percent]
**1**	UK Million Women Study [18]	**1,084,110**	**50–64**	**2.6**	**9364**	**0.86%**
**2**	Danish Nurses Health [19]	**10,874**	**>44**	**6**	**244**	**2.24%**
**3**	Melbourne Postmenopausal [20]	**13,444**	**40–69**	**10**	**336**	**2.50%**
**4**	Finnish Registry ERT [21]	**110,980**	**>50**	**8**	**2171**	**1.96%**
**5**	Finnish Registry E&P [22]	**221,551**	**>50**	**11**	**6211**	**2.80%**
**6**	French Cohort [23]	**3175**	**>50**	**13**	**105**	**3.31%**
**7**	WHI I Prempro [24]	8506	**50–79**	**5.2**	**166**	**1.95%**
**7**	WHI I Placebo [24]	8102	**50–79**	**5.2**	**124**	**1.53%**
**8**	WHI II Prempro [25]	5310	**50–79**	**7.1**	**104**	**1.96%**
**8**	WHI II Placebo [25]	5429	**50–79**	**7.1**	**133**	**2.45%**
**9**	Sweden: The Gothenburg Breast Screening Trial [26]	**51,611**	**39–59**	**14**	**1509**	**2.92%**
**10**	UK Trial of Early Detection of Breast Cancer [10]	**39,773**	**45–64**	**7**	**459**	**1.15%**
**11**	Australia Record Review of Postmenopausal Women [27]	**508**	**35–84**	**5.8**	**7**	**1.38%**
**12**	Osteoporosis Fracture Study [28]	**9704**	**>65**	**3.2**	**117**	**1.21%**
**13**	Italy ORDET [29]	**4040**	**40–69**	**3.5**	**25**	**0.62%**
**14**	NYU Postmenopausal [30]	**7063**	**35–65**	**5.5**	**121**	**1.71%**
**15**	US Breast Cancer Demonstration Detection Program [31]	**283,222**	**40–93**	**3.5**	**4275**	**1.51%**
**16**	Sweden-Malmo [32]	**42,283**	**45–69**	**25**	**2316**	**5.47%**
**17**	Norwegian Cohorts [33]	**229,256**	**50–64**	**6**	**3997**	**1.74%**
**18**	Swedish Two-Country Trial: Active Screened Group [34]	**77,052**	**40–74**	**7**	**1398**	**1.81%**
**19**	Canadian National Breast Screening Study [35]	**89,434**	**40–59**	**7**	**1332**	**1.49%**
	Total Numbers	**2,305,427**			**34,514**	
	**Mean Breast Cancer Incidence Calculation**		**34,514/2,205.427 ————→**			**1.50%**

### Analysis of All Studies

On average, the cumulative incidence rate of a first case of invasive breast cancer increased by 0.20% per year (95% CI: 0.17%, 0.23%; p < 0.01; R^2^ = 0.90). The percentage of women who remained disease free after 25 years of follow-up averaged 94.55% (95% CI: 93.97, 95.13).

**Fig 2:**
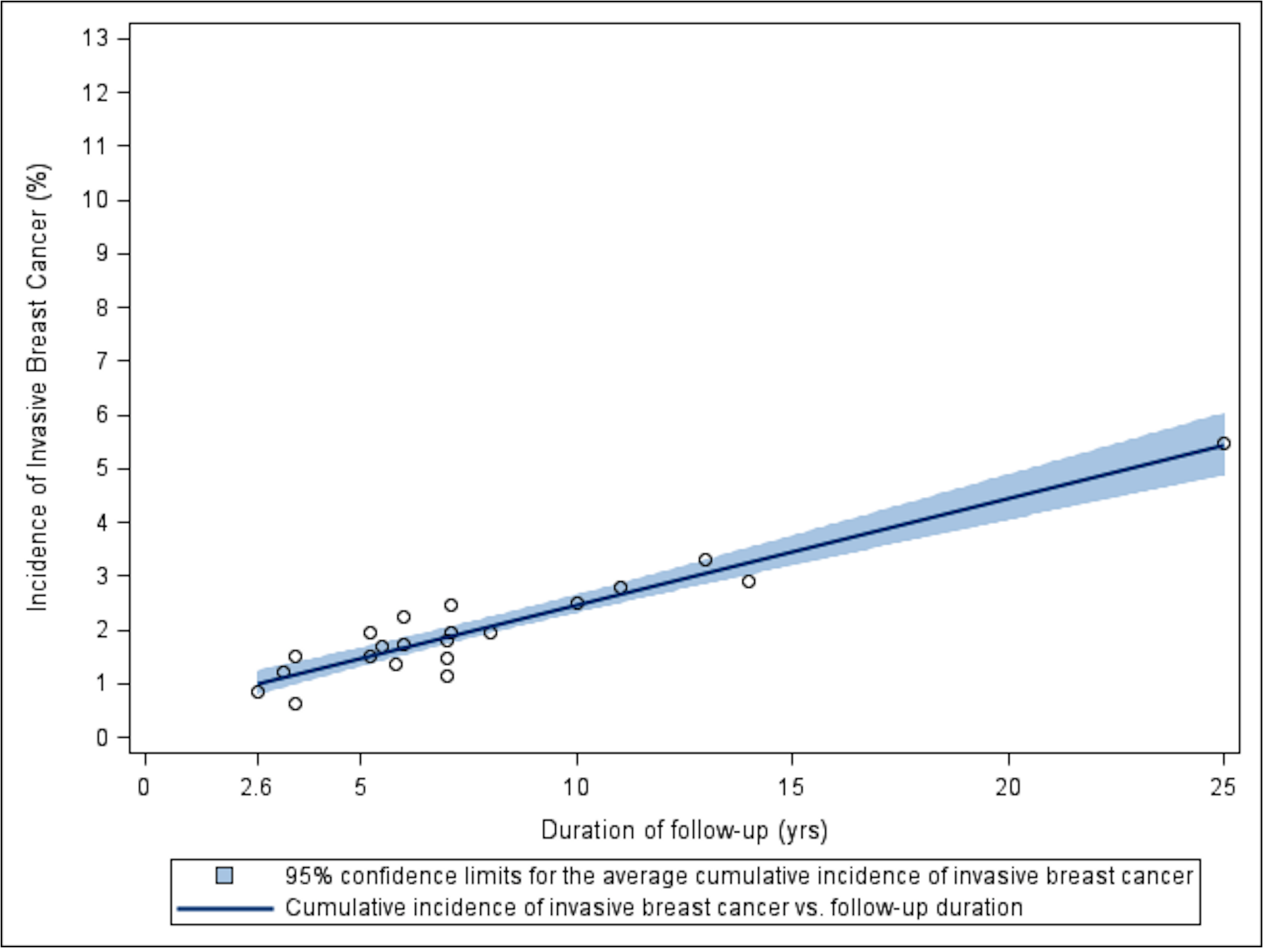
Scatterplot and regression with 95% CI for the relationship between cumulative incidence of invasive breast cancer and duration of follow-up from the 19 studies of peri/postmenopausal women. Based on all 19 studies, each plotted point is located at the intersection of the study duration and its cumulative incidence of invasive breast cancer. To focus attention on the 13% lifetime risk estimate currently advertised, the maximum of the X-axis is set at 13%.

Fig 2 illustrates the linear trend in the relationship between the cumulative incidence of first invasive breast cancer and the duration of follow-up among peri/postmenopausal women who had no history of invasive breast cancer at study enrollment.

The linear relationship demonstrated in the graph indicates that least squares regression is an appropriate statistical method to estimate the average percentage of regularly monitored women who remained breast cancer-free. Of note is the very gradual annual increase in cumulative incidence. Model diagnostics [36] suggested that the cumulative incidence rate of invasive breast cancer observed in the UK Trial of Early Detection of Breast Cancer (Study 10) [10] was lower than expected based on the general trend the rest of the studies suggested. This could be due to UK study’s continued enrollment of a disproportionate number of younger women (45 to 49 years of age).

The Sweden-Malmo study (Study 16) [32] was identified as a potentially influential observation because follow-up continued for 25 years, which was considerably longer than the next longest study (14 years). We conducted a sensitivity analysis by removing this study, and found that the study did not have a statistically or clinically relevant effect on the results. After we removed the Sweden-Malmo study, the average increase in the cumulative incidence rate of a first case of invasive breast cancer remained 0.20% per year (95% CI: 0.14%, 0.25%; p < 0.01; R^2^ = 0.76). Interim results of the Sweden-Malmo study were consistent with those of shorter studies.

**Fig 3:**
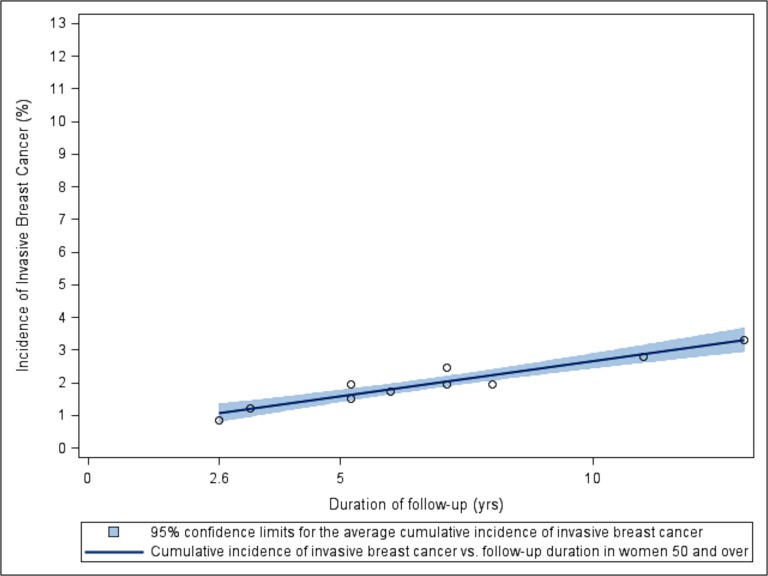
Scatterplot and regression with 95% CI for the relationship between cumulative incidence of invasive breast cancer and duration of follow-up from the 8 studies in which all women were at least 50 years old at enrollment. Regression line is based on 8 studies: #1 [18], 4 [21], 5 [22], 6 [23], 7 [24], 8 [25], 12 [28], 17 [33]. Each plotted point is located at the intersection of the duration of study and its cumulative incidence of invasive breast cancer.

When we restricted our attention to the 1,686,123 women in the 8 studies in which women were at least 50 years old at enrollment we also saw a linear relationship between cumulative incidence of first invasive breast cancer and years of follow up (Fig 3). The resulting least squares regression model was consistent with the general trend of the entire group of 19 studies.

#### Validity of Least Squares Linear Regression Model

Model diagnostics indicate that a least squares linear regression model is an appropriate analytic approach to quantify the effect of follow-up duration on the cumulative incidence of a first invasive breast cancer. We assumed that the studies we considered were independent because the same women were not included in multiple studies, and the studies we analyzed were not directly related. Fig 2 shows a linear relationship between the cumulative incidence of a first invasive breast cancer and follow-up duration. The residual plots also do not show any distinct fanning in/fanning out patterns or non-linear trends, so homogeneity and linearity can reasonably be assumed. Finally, a normal probability plot of the residuals appears fairly linear; this suggests that the normality assumption is met.

#### Analysis of Subgroup Studies: Women at Least 50 at Enrollment

Cumulative incidence rate of a first case of invasive breast cancer increased by an average of 0.22% per year among women who were enrolled in the study and at least 50 years old (95% CI: 0.16%, 0.27%; p < 0.001; R^2^ = 0.91). An average of 97.34% of the women in these studies remained disease free after 10 years of routine follow-up (95% CI: 97.10, 97.58). (Fig 3)

In this subgroup analysis, model diagnostics identified the French Cohort Study (Study 6) [23] as a potentially influential observation; however, the cumulative incidence of observed invasive breast cancer was consistent with the general trend of the other studies. The placebo group of the WHI II study (Study 8) [25] also yielded an incidence rate of invasive breast cancer higher than expected given the general trend of the studies we considered. Notably, every woman in this WHI study subgroup had undergone a hysterectomy, all were postmenopausal, and none were provided hormonal therapy.

#### Analysis of Subgroup Studies: Women at Enrollment either 50 or Older, or Surgically Menopausal

The cumulative incidence rate of a first case of invasive breast cancer increased by an average of 0.23% per year (95% CI: 0.18%, 0.28%, p< 0.0001, R^2^ = 0.88) among women who were either 50 years old or surgically menopausal when they enrolled in the study.

**Fig 4:**
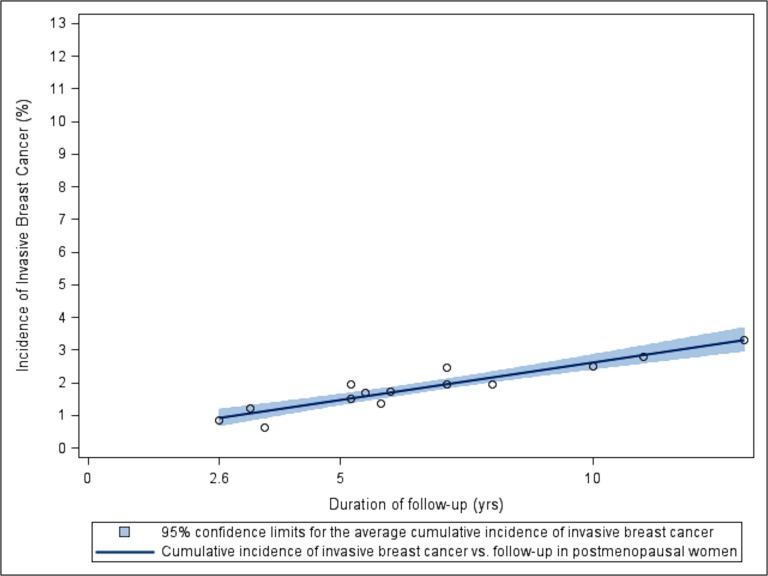
Scatterplot and regression with 95% CI for the relationship between cumulative incidence of invasive breast cancer and duration of follow-up in the 12 studies of women who were 50 years old or older or surgically menopausal at enrollment. Regression line is based on 12 studies: #1 [18], 3 [20], 4 [21], 5 [22], 6 [23], 7 [24], 8 [25], 11 [27], 12 [28], 13 [29], 14 [30], 17 [33]. Each plotted point is located at the intersection of the duration of study and its cumulative incidence of invasive breast cancer.

Fig 4 also shows a linear relationship between cumulative incidence of first invasive breast cancer and years of follow up for those studies in which women were at least 50 years old or surgically menopausal at enrollment. This subset of 12 studies included 1,711,178 women.

In this subgroup analysis, the French Cohort Study was again identified as a potentially influential observation via model diagnostics. Again, the observed cumulative incidence rate was consistent with the general trend established by the other studies. The observed cumulative incidence of invasive breast cancer in the Italy ORDET Study (Study 13) [29] was lower than expected, given the general trend the other studies exhibited. Since the potential for frequent overdiagnosis of breast cancer from mammogram screening is high, it is unsurprising that the ORDET study reported a relatively low incidence rate because their data were monitored from detailed medical records and cancer registries.

## Discussion

Based on the data analyzed from the 19 published studies of 2,305,427 asymptomatic peri/postmenopausal women, over the next 25 years of their lives, the cumulative incidence of a first invasive breast cancer is ~5%. Invasive breast cancer rates should be even lower for women who do not opt for mammograms because unscreened women are not overdiagnosed, and because *in situ* lesions, which might never cause harm, remain undetected. Incidence should be lowest for asymptomatic women whose health habits reduce risk [37].

The major strength of this analysis is that it used data from published studies that screened for breast cancer in samples of known size and duration of follow up. Our selection criteria ensured that the data did not rely on estimated population sizes, and did not count a single woman multiple times. We avoided pooling data for disparate groups by using linear regression to analyze the data, instead of meta-analysis. Because the 19 studies were independently designed, results did not depend on preconceived notions. We could calculate the total cumulative incidence of first invasive breast cancer for 2,305,427 women by using prospectively gathered data.

The CDC lifetime risk estimate of 13% is derived from data reported by the Surveillance, Epidemiology, and End Results (SEER) Program [12]. SEER Cancer Statistics Review 1975–2011 indicates that, at age 40 the risk of invasive breast cancer with 20-year follow-up is 4.67%, at 50 it is 5.56%, and at 60 it is 6.89%. But these estimates rely on registry data, so they are subject to both population underestimates and overcounting women with prior breast cancer, which may explain why SEER 20-year estimates increase by 19% between age 40 and 50, and by 24% between age 50 and 60. If they were adjusted for these potential sources of incidence overestimation, the SEER data would roughly parallel our results.

Earlier studies suggested that widespread screening, and its promise of early treatment for breast cancer, reduced mortality for the screened group below that of groups who were not screened [34], but these benefits were modest. The most direct comparison available is in the Malmo study where, after 25 years of follow-up, 1% of the women who were repeatedly screened in the early years of the program had died from breast cancer, while 1.29% of those who had not been repeatedly screened had died from breast cancer [32]. More recently, a close examination of apparently beneficial reductions in mortality rate [34] showed that mortality rate among women with a breast cancer diagnosis was only 10% lower in screened women than in unscreened women [8, 38]. By 2012, in Canada, the first randomized controlled trial of annual, protocol-trained clinical breast exam vs. clinical exam plus mammogram *showed no additional benefit of mammogram screening and no reduction in mortality with added mammography* [35]. Screened women had, however, more biopsies, more extensive surgeries, more radiation, and more drug treatments, all without a survival benefit. In 2013, the Cochrane Collaboration analysis of screening for breast cancer published a comprehensive analysis of quality and results of the major mortality outcomes in large screening trials. They concluded: “The studies which provided the most reliable information showed that screening did not reduce breast cancer mortality” [1]. In 2014, the Canadian Study published its long-term mortality data from breast cancer and showed the same percentage (1.1%) of women had died from breast cancer in each group after 25 years. Adding mammography to the protocol trained clinical breast examination did not reduce mortality from breast cancer [39].

Potential harms of widespread screening need to be weighed against benefits, and need to be clearly explained to women so that they can grant informed consent [40]. Harms include stress from fear, and complications from biopsies and follow-up testing procedures for findings that ultimately turn out to be benign or even non-existent. Long lists of adverse radiation-treatment and drug effects on well being, bone, cardiovascular, and sexual function, as well as the cumulative radiation exposure of repetitive mammograms, are the consequences of overdiagnosis [5–7]. Among enrollees in the Women’s Health Initiative Study, 38% were called back for another screen and subsequent work-up, but <2.5% received invasive breast cancer diagnoses [24, 25]. Economic harms from excessive medical costs and lost work are also substantial.

Limitations to our study include participant representativeness and impact of potentially cancer-inducing catastrophes. For example, the 1986 Chernobyl accident doubled the rate of breast cancer after 10 years in nearby regions [41, 42] and the wind that drove the rains that followed brought substantial radioactive fallout into Sweden. This may have *elevated* our trend line, since we included 2 studies conducted in Sweden during this critical period (9 and 16) [26, 32]. Women who are screened may also differ from the general population; they may be more educated, have health insurance, and/or have a family member who has had breast cancer. It is also possible that the studies we included reflect publication bias, or that the women’s concerns about their risk of breast cancer influenced their decision to enroll in the studies. Likewise the peer-reviewed literature may have reported a higher incidence of breast cancer because it represents only women who agreed to be screened. But, populations at higher risk due to family history or environmental radiation exposure, should trend our incidence calculations up, and not down. Publication bias, non-response bias, or selective outcome reporting due to increased propensity for enrollment would also be likely to raise breast cancer rates. So it is possible that the unbiased incidence would be even lower than we found.

Weight gain, inadequate exercise, excess alcohol and inadequate solar radiation generating vitamin D deficiencies are the 4 major risks factors well documented to increase the risk of breast cancer. Hormonal changes as a consequence of menopause or certain hormonal therapy regimens are also associated with increased risk. These topics are reviewed further (See S4 Text) with greater detail on hormones available [37].

By including qualified studies from 10 different countries, participants should theoretically be heterogeneous with respect to dietary habits, solar exposure, exercise, and alcohol habits. Since low risk patients were never specifically selected for any of the 19 studies, the potential presence of some of these risk factors among participants suggests that those women who manage their exposures to these recognized risks may have an even lower incidence than calculated.

Conspicuously, a 25-year follow-up for many menopausal women amounts to the balance of their lives. Therefore, for many postmenopausal women, we find their remaining, lifetime likelihood of a diagnosis of breast cancer is under ~5%.

Based on these studies of more than 2 million women, each year 99.75% of peri/postmenopausal woman without a known history of breast cancer are expected to remain free of invasive breast cancer diagnosis; 95% will not be diagnosed with an invasive breast cancer during 25 years of follow-up. Given the impact of overdiagnosis, together with the lack of evidence that mammography screening actually does reduce mortality rates, and the potential harms that may result from screening, we conclude that asymptomatic women with no breast cancer history and their physicians may reasonably decide against mammogram screening.

## Supporting Information

S1 File Sample Filemaker Card Reference Inventory from Chen 2006. (PDF)

S2 File Sample Filemaker single record of Chen 2006. (PDF)

S1 Table The Prisma Check List. (DOC)

S2 Table Steps 1 and 2 and 3 of the Records Search. (XLSX)

S1 Text Additional information about these 4009+69 papers. (DOCX)

S2 Text Sample Microsoft word record of Chen 2006. (DOC)

S3 Text Breast Cancer Incidence Drops at Rescreens. (DOCX)

S4 Text Risk Factors for Breast Cancer. (DOCX)
