# Correction: Invasive Breast Cancer Incidence in 2,305,427 Screened Asymptomatic Women: Estimated Long Term Outcomes during Menopause Using a Systematic Review

**DOI:** 10.1371/journal.pone.0135432

**Published:** 2015-08-07

**Authors:** Winnifred Cutler, Regula Bürki, James Kolter, Catherine Chambliss, Erika Friedmann, Kari Hart

There is an error in Table 2. The trial name, WHI II Prempro, in the ninth row is incorrect. The correct trial name is: WHI II Premarin. Please see the correct Table 2 here.

**Table 2:** Percent screened Peri/Postmenopausal Women Diagnosed with 1st Incident Invasive Breast Cancer in 19 published Studies.

#	Trial	Number of Women	Baseline Age	Years Studied	Number with Breast Cancer	Incidence [Percent]
**1**	UK Million Women Study [18]	**1,084,110**	**50–64**	**2.6**	**9364**	**0.86%**
**2**	Danish Nurses Health [19]	**10,874**	**>44**	**6**	**244**	**2.24%**
**3**	Melbourne Postmenopausal [20]	**13,444**	**40–69**	**10**	**336**	**2.50%**
**4**	Finnish Registry ERT [21]	**110,980**	**>50**	**8**	**2171**	**1.96%**
**5**	Finnish Registry E&P [22]	**221,551**	**>50**	**11**	**6211**	**2.80%**
**6**	French Cohort [23]	**3175**	**>50**	**13**	**105**	**3.31%**
**7**	WHI I Prempro [24]	8506	**50–79**	**5.2**	**166**	**1.95%**
**7**	WHI I Placebo [24]	8102	**50–79**	**5.2**	**124**	**1.53%**
**8**	WHI II Premarin [25]	5310	**50–79**	**7.1**	**104**	**1.96%**
**8**	WHI II Placebo [25]	5429	**50–79**	**7.1**	**133**	**2.45%**
**9**	Sweden: The Gothenburg Breast Screening Trial [26]	**51,611**	**39–59**	**14**	**1509**	**2.92%**
**10**	UK Trial of Early Detection of Breast Cancer [10]	**39,773**	**45–64**	**7**	**459**	**1.15%**
**11**	Australia Record Review of Postmenopausal Women [27]	**508**	**35–84**	**5.8**	**7**	**1.38%**
**12**	Osteoporosis Fracture Study [28]	**9704**	**>65**	**3.2**	**117**	**1.21%**
**13**	Italy ORDET [29]	**4040**	**40–69**	**3.5**	**25**	**0.62%**
**14**	NYU Postmenopausal [30]	**7063**	**35–65**	**5.5**	**121**	**1.71%**
**15**	US Breast Cancer Demonstration Detection Program [31]	**283,222**	**40–93**	**3.5**	**4275**	**1.51%**
**16**	Sweden-Malmo [32]	**42,283**	**45–69**	**25**	**2316**	**5.47%**
**17**	Norwegian Cohorts [33]	**229,256**	**50–64**	**6**	**3997**	**1.74%**
**18**	Swedish Two-Country Trial: Active Screened Group [34]	**77,052**	**40–74**	**7**	**1398**	**1.81%**
**19**	Canadian National Breast Screening Study [35]	**89,434**	**40–59**	**7**	**1332**	**1.49%**
	Total Numbers	**2,305,427**			**34,514**	
	**Mean Breast Cancer Incidence Calculation**		**34,514/2,205.427 ————→**			**1.50%**
