# Supplementary material for: Invasive Breast Cancer Incidence in 2,305,427 Screened Asymptomatic Women: Estimated Long Term Outcomes during Menopause Using a Systematic Review
Source: PLoS One. 2015 Jun 24;10(6):e0128895. doi: 10.1371/journal.pone.0128895 (PMC4479875; doi:10.1371/journal.pone.0128895)
Supplement: S1 File — (PDF) [file pone.0128895.s001.pdf]

Card Reference Inventory HX2

	PrimaryTopic	Year	Journal	Authors	Article Title	WBC Card
1		2007	FertilSteril	Abdallah MA,	Effects of the	
2	Uterus	2003	ObstetGynecol	Al-Fozan H, Tulandi T	Left lateral predisposition	2/17/
3	Uterus	2006	ObstetGynecol	Al-Sunaidi M, Tulandi T	Adhesion-related bowel	1/19/
4	Incontinence	2005	Climacteric	Albertazzi P & Sharma S	Urogenital effects of	3/9/2
5		1999	Lancet	Alexander FE, Anderson	14 years of follow-up	
6		2008	Cancer	Andersen BL, Yang	Psychologic	
7	HRT: Breast	2006	Menopausal	Archer DF	Hormone therapy and	10/6/
8	Altern med &	2004	Breast Can	Atkinson C, Warren RML,	Red clover-derived	2/10/
9	Altern med &	1998	FertilSteril	Awoniyi CA, Roberts D,	Reproductive sequelae in	11/3/
10	Sex & Sex	2005	FertilSteril	Aziz A, Brannstrom M,	Perimenopausal androgen	9/7/2
11	Uterus	2006	FertilSteril	**Ballard K, Lowton K,**	**Balancing the risks and**	1/19/
12	HRT: Breast	2001	Breast Cancer	Banks E, Beral V,	Comparison of various	6/17/
13	Uterus	2004	ObstetGynecol	Benard VB, Eheman CR,	Cervical screening in the	2/6/2
14	HRT: Breast	2003	Lancet	Beral V	Breast cancer and	6/11/
15	HRT: Breast	1997	Lancet	Beral V, Bull D, Doll R,	Breast cancer and	8/13/
16		1996	J Natl Cancer	Berrino F, Muti P,	Serum sex hormone	
17	Cognitive Function	2002	Psychneuroendo	Bethea CL, Mirkes SJ, Su	Effects of oral estrogen,	5/10/
18	HRT	2005	Arch Women	Birkhaeuser M	Invited editorial: The	1/11/
19	Altern med &	2004	Climacteric	Bjarnason NH, Jorgensen	Smoking reduces breast	1/28/
20	Uterus	2004	FertilSteril	Blumenfeld Z	Hormonal suppressive	1/12/
21	Pharmacokinetics	2004	Menopause	Boothby LA, Doering PL,	Bioidentical hormone	11/12
22	HRT: Breast	1995	J Natl Cancer	Boyd NF, Byng JW, Jong	Quantitative classification	8/25/
23	Bone Health	2003	Nature	Boyle WJ, Simonet S,	Osteoclast differentiation	5/9/2
24	HRT: Breast	2003	Maturitas	Braendle, W. (	"The Million Women	10/4/
25		2007	Menopause	Burger HG	Should testosterone	
26	HRT: Breast	2001	ObstetGynecol	Bush TL, Whiteman M,	Hormone replacement	12/15
27	Sex & Sex	2005	ObstetGynecol	Buster JE, Kingsberg SA,	Testosterone patch for	9/11/
28	CVH	2006	Menopause	Cagnacci A, Tonie AD,	Cyclic progestin	6/27/
29	Altern med &	2003	Psychoneur	Carlson LE, Speca M,	Mindfulness-based stress	2/28/

Card Reference Inventory HX2

	PrimaryTopic	Year	Journal	Authors	Article Title	WBC Car
30	Altern med &	2004	Menopause	Carpenter JS, Gilchrist	Hot flashes, core body	3/30/
31		2006	FertilSteril	Casini ML, Marelli G,	Psychological	4/26/
32		1999	Ann Intern Med	Cauley JA, Lucas	Elevated serum	
33	Uterus	2003	ObstetGynecol	Cha D, Khosrotehrani K,	Cervical cancer and	1/11/
34	Uterus	2005	AmJ ObGyn	Chalas E, Costantino JP,	Tamoxifen and benign	3/10/
35	Uterus	2006	ObstetGynecol	Chan JK, Cheung	Patterns and	1/19/
36	Uterus	2006	ObstetGynecol	**Chan K, Cheung MIK,**	**Patterns and**	1/19/
37	Altern med &	2006	Menopause	Chang CJ, Chiu JH,	Si-Wu-Tang and its	12/5/
38	HRT: Breast	2002	JAMA	Chen CL, Weiss NS,	Hormone replacement	7/22/
39	HRT: Breast	2002	JAMA	Chen CL, Weiss NS,	Hormone replacement	7/22/
40	HRT: Breast	2002	JAMA	Chen CL, Weiss NS,	Hormone replacement	7/22/
41	HRT: Breast	2006	Arch InternMed	Chen WY, Manson JE,	Unopposed estrogen	9/29/
42	HRT: Breast	2004	Lancet	Chlebowski RT, Col N	Menopausal hormone	10/3/
43	HRT: Breast	2003	ACOG Clin Rev	Chlebowski RT, Hendrix	Influence of estrogen plus	
44	HRT: Breast	2003	J Natl Cancer	Cho E, Spiegelman D,	Premenopausal fat intake	11/27
45	Uterus	2004	FertilSteril	Cicinelli E, de Ziegler D,	First uterine pass effect"	9/8/2
46	HRT: Breast	2004	Climacteric	Clarke RB	Human breast cell	9/25/
47	HRT	2002	Climacteric	Clarke SC, Kelleher J,	A study of hormone	1/19/
48	HRT: Breast	2001	FertilSteril	Colacurci N, Fornaro F,	Effects of a short-term	5/13/
49	HRT: Breast	1995	N Engl J Med	Colditz GA, Hankinson	The use of estrogens and	7/20/
50			12(4): 436	Conde DM, Pinto-Neto	Menopause symptoms	10/9/
51	HRT: Breast	2004	FertilSteril	Conner P, Svane G,	Mammographic breast	8/10/
52	HRT: Breast	2005	BMJ	Coombs NJ, Taylor R,	Hormone replacement	10/5/
53	Altern med &	1985	SocSciMed	Coulehan JL	Chiropractic and the	10/13
54			*Psychological*	Coyne JC, Stefanek	Psychotherapy and	
55		2009	Annals	Coyne JC, Thombs	A meta-analysis of	
56	HRT: Breast	2002	Cancer	Daling JR, Malone KE,	Relation of regimens of	7/26/
57	HRT: Breast	2003	CancerEpidem,	Daling JR, Malone KE,	Association of regimens	8/16/
58	CVH	2002	Climacteric	Davis SR, Goldstat R,	Differing effects of low-	7/11/

Card Reference Inventory HX2

	PrimaryTopic	Year	Journal	Authors	Article Title	WBC Car
59	CVH	2003	Menopause	Davis SR, Goldstat R,	Effects of aromatase	4/28/
60	Uterus	2006	Climacteric	Davy M, Oehler MK	Controversial issues:	1/23/
61	HRT: Breast	2002	Climacteric	de Lignieres B	Editorial: Effects of	9/26/
62	HRT: Breast	2002	Climacteric	de Lignieres B, de	Combined hormone	5/7/2
63	HRT: Breast	1997	Breast	Decker DA, Pettinga JE,	Hormone replacement	5/8/2
64	HRT: Breast	2003	Menopause	Decker DA, Pettinga JE,	Estrogen replacement	5/21/
65	Altern med &	2004	Psychoneur	Detillion CE, Craft TKS,	Social facilitation of	9/23/
66	HRT: Breast	2003	Climacteric	Dew JE, Wren BG, Eden	A cohort study of topical	5/17/
67	HRT: Breast	2004	Menopause	Dimitrakakis C, Jones RA,	Breast cancer incidence in	12/21
68	HRT: Breast	2003	Menopause	Dimitrakakis C, Zhou J,	A physiologic role for	5/26/
69	Altern med &	2003	Menopause	Dog TL, Powell KL,	Critical evaluation of the	10/13
70	HRT	2003	Climacteric	Domoney C, Studd WW,	Continuation of hormone	11/26
71		1996	CancerEpidem,	Dorgan JF,	Relation of	
72	HRT: Breast	1997	CancerEpidem,	Dorgan JF, Stanczyk FZ,	Relationship of serum	9/26/
73	HRT	2005	FertilSteril	Draelos ZD	Topical and oral estrogens	11/24
74	HRT: Breast	2003	Maturitas	Druckmann R	Progestins and their	8/9/2
75	HRT: Breast	2004	Climacteric	Durna EM, Heller GZ,	Breast cancer in	10/2/
76	HRT: Breast	2002	MedJ Aust	Durna EM, Wren BG,	Hormone replacement	10/2/
77	CVH	2005	Menopause	Duvernoy CS, Kulkarni	Vascular events in the	2/1/2
78		2008	J Natl Cancer	Eisen A, Lubinski J,	Hormone therapy and the	
79	HRT: Breast	2005	Climacteric	Farmer R	Invited Editorial: The	10/4/
80	HRT: Breast	1998	FertilSteril	Foidart JM, Colin C,	Estradiol and	8/9/2
81	HRT: Breast	2003	Bull Cancer	Fournier, A., F. Clavel-	"[Menopause hormonal	
82	HRT: Other	2003	Menopause	Frank E, Elon L	Clinical and personal	11/24
83		2006	Am J Clin	Galeone C, Pelucchi	Onion and garlic use	
84		2006	Climacteric	Gambrell RD,	Moderate dosage	11/28
85		1992	Am J Epide	Garland CF,	Sex hormones and	
86	Altern med &	2006	Climacteric	Geller S, Studee L.	Soy and red clover	12/5/
87	HRT: Breast	2006	Menopause	Gertig DM, Fletcher AS,	Hormone therapy and	10/2/

Card Reference Inventory HX2

	PrimaryTopic	Year	Journal	Authors	Article Title	WBC Car
88	Endocrinology (not	2003	Psychneuroendo	Gold SM, Zakowski SG,	Stronger endocrine	
89	CVH	2004	ObstetGynecol	Grady D, Ettinger B,	Safety and adverse	10/26
90	HRT: Breast	2002	Cancer	Grant WB	An ecologic study of	9/25/
91	HRT: Breast	2002	Cancer	Grant WB	An estimate of premature	9/22/
92	Sex & Sex	2001	Menopause	Greendale GA, Petersen	Factors related to sexual	9/8/2
93	Uterus	2003	FertilSteril	Grigorieva V, Chen-Mok	Use of a levonorgestrel-	11/12
94	Incontinence	1997	N Engl J Med	Grodstein F, Stampfer	Postmenopausal hormone	2/13/
95	HRT: Breast	1999	ObstetGynecol	Guidozzi, F.	Award article: Estrogen	6/16/
96	Cognitive Function	2006	Psychoneur	Hammerfald K, Eberle C,	Persistent effects of	4/10/
97	HRT	2005	Climacteric	Harman SM, Brinton EA,	Invited Editorial: KEEPS:	12/5/
98	Sex & Sex	2004	ObstetGynecol	Hartmann KE, Ma C,	Quality of life and sexual	9/7/2
99	HRT: Breast	2004	Radiology	Harvey JA, Bovbjerg VE	Quantitative assessment	8/12/
100	HRT	2004	Radiology	Harvey JA, Bovbjerg VE	Quantitative assessment	8/12/
101	HRT: Breast	2005	Climacteric	Harvey JA, Scheurer C,	Hormone replacement	11/30
102	Bone Health	2003	AmJ ClinNutr	Heaney RP	Long-latency deficiency	4/15/
103		1994	Cancer Detect	Helzlsouer KJ,	A prospective study	
104		2007	Menopause	Hofling M,	Testosterone inhibits	
105	HRT: Breast	1999	JCEM	Hofseth LJ, Raafat AM,	Hormone replacement	6/18/
106	Bone Health	2004	AmJ ClinNutr	Holick MF	Vitamin D: importance in	4/18/
107	HRT: Breast	2004	Lancet	Holmberg L,	HABITS (hormonal	10/3/
108	Uterus	2003	ObstetGynecol	Hsu K, Huang S, Hsiao J,	Clinical significance of	2/6/2
109	HRT	2002	JAMA	Hulley S, Furberg C,	Noncardiovascular disease	1/12/
110	CVH	1998	JAMA	Hulley S, Grady D, Bush	Randomized trial of	5/7/2
111	HRT: Breast	2002	AnnalsInternal	Humphrey LL, Helfand M,	Breast cancer screening: a	12/15
112	Uterus	2005	FertilSteril	Hurst BS, Matthews ML,	Laparoscopic	3/14/
113		2005	CancerEpidem,	Il'yasova,D, Colbert LH,	Circulating levels of	
114		2000	AmJ ClinNutr	Jacobs DR, Pereira	Fiber from Whole	
115	Uterus	2003	FertilSteril	Jadoul P, Donnez J	Conservative treatment	2/16/
116		2009	Obstet Gynecol	Jick SS, Hagberg	Postmenopausal	

Card Reference Inventory HX2

	PrimaryTopic	Year	Journal	Authors	Article Title	WBC Car
117	Uterus	2004	FertilSteril	Jirecek S, Lee A, Pavo I,	Raloxifene prevents the	2/27/
118	HRT	2005	ObstetGynecol	Johnson SR, Ettinger B,	Uterine and vaginal	12/29
119		2001	*Proc. Nutr. Soc.*	Kaaks R, Lukanova A	Energy balance and	
120	HRT: Breast	2006	Menopause	Kaunitz AM	Editorial: Hormone	10/5/
121	HRT: Breast	2003	AnnalsInternal	Kerlikowske K, Smith-	Evaluation of abnormal	12/17
122	Uterus	2003	FertilSteril	Keshavarzi A, Vaezy S,	Treatment of uterine	9/14/
123		2002	J Natl Cancer	Key T, Appleby P,	Endogenous sex	
124	CVH	2005	FertilSteril	Klaiber EL, Vogel W,	A critique of the Women's	12/8/
125	Altern med &	2002	Am J Clin	Knekt P, Kumpulainen J,	Flavonoid intake and risk	1/26/
126	Altern med &	2004	JAMA 292:1	Knoops KT, de Groot LC,	Mediterranean diet,	1/24/
127	HRT: Breast	2004	Abstracts of the	Kraemer EA Seeger H,	Possible influence of	9/21/
128	HRT: Breast	2005	Menopause	Kraemer EA, Seeger H,	The effects of	9/29/
129	Altern med &	2004	JAMA	Kreijkamp-Kaspers S,	Effect of soy protein	3/28/
130	Pharmacokinetics	2005	Cancer	Kuhl H	Pharmacology of	1/11/
131		2007	Climacteric	Kuhl H, Wiegratz I	Can 19-	10/7/
132	HRT	2003	FertilSteril	Kukuvitis A, Kourtis A,	Differential effects of	12/8/
133	HRT	2002	JAMA	Lacey JV, Mink PJ, Lubin	Menopausal hormone	1/13/
134	HRT	2005	Climacteric	Lauritzen C	Letter to the Editors:	1/12/
135		2009	BMJ	Lee AHS, Ellis IO	Asymptomatic breast	
136	HRT: Breast	2004	AmJ ClinOncol	Legorreta AP, Chernicoff	Diagnosis, clinical staging,	12/21
137	Cognitive Function	2001	Psychneuroendo	Legros J	Inhibitory effect of	8/13/
138		2007	N Engl J M	Lehman CD, Gatsonis C,	MRI evaluation of the	
139	HRT: Breast	2003	JAMA	Li CI, Malone KE, Porter	Relationship between long	7/28/
140		2008	Breast Cancer	Lietzmann MF, Moore	Prospective study of	
141	Uterus	2004	FertilSteril	Lin PC, Thyer A, Soules	Intrapretative ultrasound	12/28
142	Uterus	2006	ObstetGynecol	Lochner D, Brubaker KL	Incidence of malignancy in	1/17/
143	Incontinence	2006	FertilSteril	Long C. Liu C, Hsu S,	A randomized	3/7/2
144	HRT: Breast	2003	Menopause	Longcope C	Editorial: Androgens,	5/26/
145	HRT: Breast	2006	FertilSteril	Lundstrom E, Soderqvist	Digitized assessment of	9/21/

Card Reference Inventory HX2

	PrimaryTopic	Year	Journal	Authors	Article Title	WBC Car
146		2009	Obstet Gynecol	Lyytinen H, Pukkala	Breast cancer risk in	
147	HRT: Breast	2006	ObstetGynecol	Lyytinen, H, Pukkala E,	Breast Cancer risk in	
148	CVH	2003	Atherosclerosis	Mack WJ, Hameed AB,	Does elevated body mass	4/5/2
149	HRT: Breast	1999	Int J Cancer	Magnusson C, Baron JA,	Breast-cancer risk	7/20/
150		1995	Rockville	Maklan CW, Else B.	Treatment of common	
151	HRT	2006	Menopause	Manson JE, Bassuk	Personal Perspective:	11/28
152	HRT: Breast	2002	N Engl J Med	Marchbanks PA,	Oral contraceptives and	8/31/
153	CVH	2005	Arch InternMed	Margolis KL, Manson JE,	Leukocyte count as a	1/3/2
154	CVH	2004	Annals	Masters KS, Hill RD,	Religious orientation,	6/29/
155		2008	Nature Reviews	McTiernan A	Mechanisms linking	
156		2004	CancerEpidem,	McTiernan A, Tworoger	Effect of exercise on	
157	CVH	1999	N Engl J Med	Mendelsohn ME, Karas	The protective effects of	4/27/
158	HRT: Breast	2002	AnnalsInternal	Miller AB To T, Baines	The Canadian national	9/20/
159	HRT: Breast	2002	BJOG	Million Women Study	Patterns of use of	6/16/
160	HRT: Breast	2004	J Natl Cancer	Missmer SA, Eliassen	Endogenous	9/26/
161	Carbohydrate	2005	Menopause	Misso M, Jang C, Adams	Adipose aromatase gene	11/28
162	Uterus	2005	ObstetGynecol	Moalli PA, Shand SH,	Remodeling of vaginal	2/27/
163	Cognitive Function	2005	PNEC	Mook D, Felger J, Graves	Tamoxifen fails to affect	11/16
164	HRT: Breast	2003	Climacteric	Mueck AO, Seeger H,	Comparison of the	8/3/2
165		2003	European J	Muller M, den	Endogenous sex	
166	HRT: Breast	1991	Molecular	Musgrove EA, Lee CS,	Progestins both stimulate	8/3/2
167	HRT	2004	Climacteric	Naftolin F, Schneider	Guidelines for hormone	11/30
168	HRT: Other	2004	FertilSteril	Naftolin F, Taylor HS,	The Women's Health	1/30/
169	CVH	2005	Health,	National Center for	Table 29 (page 1 of 4).	6/27/
170	HRT	2006	Menopause	Naunton M, Hadithy	Estradiol gel: review of	11/27
171	HRT: Breast	2003	Climacteric	Neves-e-Castro M	Some comments on	10/6/
172		1987	Br J Cancer	Nielsen M, Thomsen JL,	Breast cancer and atypia	
173	HRT: Breast	2003	ObstetGynecol	Nikander E, Kilkkinen A,	A randomized placebo-	5/20/
174	Altern med &	2005	FertilSteril	Nikander E, Rutanen EM,	Lack of effect of	11/8/

Card Reference Inventory HX2

	PrimaryTopic	Year	Journal	Authors	Article Title	WBC Car
175	HRT: Breast	2003	Cancer	Norman SA, Berlin JA,	Combined effect of oral	8/23/
176	Bone Health	2002	Menopause	Notelovitz M, John VA,	Effectiveness of Alora	5/31/
177	Incontinence	2005	ObstetGynecol	Nygaard I, Girts T, Fultz	Is urinary incontinence a	3/8/2
178	HRT: Breast	2002	Lancet	Nystrom L, Andersson I,	Long-term effects of	9/20/
179	Uterus	2003	ObstetGynecol	O'Hanlan KA, Lopez L,	Total laparoscopic	3/15/
180	HRT: Breast	2001	JNCI	O'Meara ES, Rossing MA,	Hormone replacement	8/24/
181	Uterus	2004	FertilSteril	Omodei U, Ferrazzi E,	Endometrial evaluation	9/2/2
182	Uterus	2005	Epidem	Ossewaarde ME, Bots	Age at menopause,	5/23/
183	Uterus	2005	Epidem	Ossewaarde ME, Bots	Age at menopause,	5/23/
184	HRT: Breast	2004	CancerEpidem,	Page JH, Colditz GA, Rifai	Plasma adrenal androgens	9/27/
185	HRT: Breast	2003	AJR	Parisky YR, Sardi A,	Efficacy of computerized	6/2/2
186	Uterus	2006	Climacteric	Parker WH, Broder MS,	Letters to the Editors:	1/23/
187	Uterus	2005	ObstetGynecol	Parker WH, Broder MS,	Ovarian conservation at	3/21/
188	Uterus	2003	FertilSteril	Payne JF, Haney AF	Serious complications of	11/25
189	Pheromones	1999	Genetica	Pearse-Pratt R,	Soluble MHC antigens and	7/20/
190	HRT	2003	FertilSteril	Penotti M, Fabio E,	Effect of soy-derived	11/2/
191	HRT	2005	FertilSteril	Phillips LS, Langer RD	Postmenopausal hormone	12/9/
192		2007	*Journal of*	Pierce,JP, Stefanick ML,	Greater survival after	
193	HRT: Breast	2006	Climacteric	Pines A, Sturdee	WHI and breast	10/5/
194		2007	Climacteric	Pines A, Sturdee	IMS reaction to	
195	CVH	2002	JAMA	Pradhan AD, Manson JE,	Inflammatory biomarkers,	4/1/2
196		2006	Int J Cancer	Pukkala E,	Breast cancer in	
197	Altern med &	2003	ObstetGynecol	Queenan JT	Smoking: the cloudy,	1/28/
198		2007	N Engl J M	Ravdin PM, Cronin KA,	The decrease in breast-	
199	CVH	2003	Climacteric	Regitz-Zagrosek V	Cardiovascular disease in	2/2/2
200	HRT: Other	2004	Menopause	Ringa V, Legare F, Dodin	Hormone therapy	11/24
201	HRT: Breast	2000	JNCI	Ross RK, Paganini-Hill A,	Effect of hormone	6/23/
202	CVH	2003	Climacteric	Rubig A	Drospirenone: a new	1/1/2
203		2007	CA Cancer J Clin	Saslow D, Boetes C,	American Cancer Society	

Card Reference Inventory HX2

	PrimaryTopic	Year	Journal	Authors	Article Title	WBC Car
204		2007	Climacteric	Sator PG, Sator MO,	A prospective,	10/20
205		2000	JAMA	Schairer C, Lubin J,	Menopausal Estrogen and	
206	HRT: Other	2001	JAMA	Schaumberg DA, Buring	Hormone replacement	11/9/
207	HRT	1996	Int J Derm	Schmidt JB, Binder M,	Treatment of skin aging	1/31/
208	HRT	2005	Climacteric	Schneider HPG, Mueck	Presidential comment:	1/13/
209	Bone Health	2002	Journal of	Schousboe JT, DeBold	Prevalence of vertebral	5/30/
210	HRT: Breast	2004	JAMA	Schwartz LM, Woloshin	Enthusiasm for cancer	12/14
211	HRT: Breast	2001	FertilSteril	Sendag F, Terek MC,	Mammographic density	5/11/
212	HRT: Breast	2005	Menopause	Shah NR, Borenstein J,	Postmenopausal hormone	9/28/
213	HRT: Breast	2004	Climacteric	Shapiro S	The Million Women Study:	10/6/
214	Sex & Sex	1998	Menopause	Shifren JL, Nahum R,	Incidence of sexual	5/26/
215	nutrition	2002	J Natl Cancer	Shin M, Holmes S,	Intake of dairy	10/25
216	Altern med &	2005	Menopause	Simoncini T, Fornari L,	Activation of nitric oxide	2/3/2
217	Pharmacokinetics	2005	Climacteric	Sitruk-Ware R	Editorial: Estrogen and	1/6/2
218	Pharmacokinetics	2004	Climacteric	Sitruk-Ware R, Husmann	Role of progestins with	1/19/
219	CVH	2003	Climacteric	Sitruk-Ware RL	Hormone therapy and the	1/30/
220		2003	CA Cancer J Clin	Smith RA, Saslow D,	American cancer	
221	HRT: Breast	2003	JAMA	Smith-Bindman R, Chu	Comparison of screening	12/20
222	Cognitive Function	2003	Psychneuroendo	Sobrinho LG, Simoes M,	Cortisol, prolactin, growth	3/9/2
223	HRT	2006	Climacteric	Soliman NF, Hillard TC	Hormone replacement	
224	Pheromones	2004	Hormones&Beha	Spencer NA, McClintock	Social chemosignals from	8/25/
225	Uterus	2005	ObstetGynecol	Spies JB, Bruno J,	Long-term outcome of	2/22/
226	HRT: Breast	2005	International J	Stahlberg C, Lynge E,	Breast cancer incidence,	9/29/
227	HRT: Breast	2004	Int J Cancer	Stahlberg C, Pedersen	Increased risk of breast	1/14/
228	HRT: Other	2005	Menopause	Stanczyk FZ	"Natural" versus	1/16/
229	HRT: Breast	1995	JAMA	Stanford JL, Weiss NS,	Combined estrogen and	8/23/
230	HRT: Breast	2006	JAMA	Stefanick M, Anderson	Effects of conjugated	10/9/
231	Uterus	2003	ObstetGynecol	Stewart EA, Gedroyc	Focused ultrasound	9/20/
232	HRT: Breast	2006	Climacteric	Sturdee DW,	Reducing the	10/6/

Card Reference Inventory HX2

	PrimaryTopic	Year	Journal	Authors	Article Title	WBC Car
233	HRT	2004	Climacteric	Sturdee DW, Rantala ML,	The acceptability of a	12/5/
234		2006	Arch InternMed	Tamimi RM,	Combined estrogen	
235	CVH	2006	Climacteric	Tanko LB, Christiansen C	Adipose tissue, insulin	6/26/
236	Uterus	2003	Radiology	Tempany CM, Stewart	MR imaging-guided	9/20/
237	HRT	2004	FertilSteril	Tempfer CB, Riener E-K,	DNA microarray-based	12/9/
238		2002	N Engl J Med	Thakar R, Ayers S,	Outcomes after total	
239	HRT: Breast	1999	Breast Cancer	The Million Women Study	The Million Women Study:	6/16/
240	Uterus	2006	FertilSteril	**The Practice**	**Treatment of pelvic**	1/19/
241		1997	Br J Cancer	Thomas HV, Key TJ,	A prospective study	
242	Altern med &	2003	JAMA 290:2	Tice JA, Ettinger B,	Phytoestrogen	2/10/
243	Uterus	2003	Menopause	Tommaselli GA, Di Carlo	Effects of bilateral	12/9/
244		1995	J Natl Cancer	Toniolo PG, Levitz M,	A prospective study	
245	Uterus	2003	Menopause	Tregon ML, Blumel JE,	The early response of the	2/20/
246	Altern med &	2003	N Engl J Med	Trichopoulou A,	Adherence to a	2/2/2
247	Altern med &	2005	ObstetGynecol	Trimble CL, Genkinger	Active and passive	2/7/2
248	Bone Health	2003	BMJ	Trivedi DP, Doll R, Khaw	Effect of four monthly	4/19/
249		1999	Lancet	UK Trial of Early	16-year mortality from	
250	Altern med &	2004	FertilSteril	Unfer V, Casini ML,	Endometrial effects of	11/3/
251	HRT	2005	Menopause	Utian WH, Speroff L,	Comparative controlled	12/30
252		2009	BMJ	Vaidya JS	Women undergoing	
253	HRT: Breast	2004	FertilSteril	Valdivia I, Campodonico	Effects of tibolone and	9/22/
254	HRT: Breast	2004	ObstetGynecol	van der Mooren, M.	The Million Women	10/6/
255	CVH	2005	Menopause	Vitale C, Cornoldi A,	Interleukin-6 and flow-	1/26/
256	HRT	2005	ObstetGynecol	Waetjen LE, Brown JS,	The effect of ultralow-	12/28
257	Uterus	2004	FertilSteril	Wallach EE, Vlahos NF	Uterine myomas: an	9/16/
258	Uterus	2002	*EuroJ Endocrin*	Wang Y, Matsuo H,	Down-regulation of	
259	Uterus	1996	N Engl J Med	Weber AM, Lee J	Use of alternative	3/1/2
260	HRT: Breast	2002	ObstetGynecol	Weiss LK, Burkman RT,	Hormone replacement	5/17/
261		1997	Ann Intern Med	Welch HG, Black WC	Using autopsy series to	

Card Reference Inventory HX2

	PrimaryTopic	Year	Journal	Authors	Article Title	WBC Car
262	Sex & Sex	1996	JSexResearch	Whipple B, Gerdes CA,	Sexual response to self-	5/7/2
263	Bone Health	2005	J Nutr	Whiting SJ, Calvo MS	Dietary recommendations	5/20/
264		2007	Psychoneur	Witek-Janusek L,	Psychologic stress,	
265		2005	*Lancet Oncol.*	Wolf S, Rubinek T	Diabetes mellitus and	
266	HRT: Breast	2004	Climacteric	Wren BG	Do female sex hormones	9/27/
267	HRT: Other	2003	Climacteric	Wren BG	Editorial: Progesterone	12/3/
268	HRT: Breast	2002	JAMA	Writing Group for the	Risks and benefits of	1/9/2
269	Uterus	2006	ObstetGynecol	Yasmeen S, Romano PS,	Incidence of cervical	1/23/
270	HRT: Breast	2003	Climacteric	Yenen MC, Dede M,	Hormone replacement	5/14/
271	CVH	2002	Climacteric	Zacharieva S,	Effect of transdermal	2/11/
272	HRT: Breast	2004	*Br J Cancer*	Zahl P-H	Overdiagnosis of	10/6/
273	HRT: Breast	2004	BMJ	Zahl P, Strand BH,	Incidence of breast cancer	12/14
274		2008	Arch InternMed	Zahl PH, Maehlen J,	The natural history of	
275	Uterus	2003	ObstetGynecol	Zekam N, Oyelese Y,	Total versus subtotal	11/26
276	Uterus	2003	FertilSteril	Zupi E, Pocek M, Dauri M,	Selective uterine artery	11/24
