# Supplementary material for: Invasive Breast Cancer Incidence in 2,305,427 Screened Asymptomatic Women: Estimated Long Term Outcomes during Menopause Using a Systematic Review
Source: PLoS One. 2015 Jun 24;10(6):e0128895. doi: 10.1371/journal.pone.0128895 (PMC4479875; doi:10.1371/journal.pone.0128895)
Supplement: S2 File — (PDF) [file pone.0128895.s002.pdf]

Card Reference List

Authors
Chen CL, Weiss NS, Newcomb P, Barlow W, White, E

Year
2002

CardRefID
Ref-A--0001

Article Title
Hormone replacement therapy in relation to breast cancer

PrimaryTopic
HRT: Breast

Related Topics: ( )
THese files are mov
so they won't open
Portal to "all fields"

TopicDetail ⑦

Journal
JAMA

Ref_Citation
JAMA 287(6):734-741

Vol.
287

Page Range
734-741

Date Initiated	1/12/05
Staffer	RN
WBC Card Date	7/22/04
Carding_Status	complete
AccuracyCheck	LB 7/20/06

	ChapterID	MS	chapter#	chapter_name_lkp		WBCArticleID
X	029-C	WHAT	11	Preserving	Ref-A-	WBC-001
X	032-C	WHAT	12	Protecting the	Ref-A-	WBC-001
X						

WBCUsedRefDetail ⑦ portal

Add New Chapter/Edit Chapters

Add ALL

H&M-fir Refs

WBC Summary

Cited by Li et al JAMA 2003 Friday, July 9, 2004 we need this one to see how they defined sequential HRT (definitions turned out to be "Women who had a different number of estrogen and progestin pills in each prescription (usually 25 e with 10 p pills) were considered sequential therapy users."
This study of enrollees of the Group Health Cooperative of Puget sound (GHC) focused on histological eval of lobular (rare but increasing) vs ductal breast cancer and HRT.
Cases: 705 of 752 women newly diagnosed with a first primary invasive breast cancer betwe 7/1/90 and 12/31/95 aged 50 to 74.
q Reference date for each case was 1 yr before the diagnosis
Controls: 692 of 752 randomly selected from GHC enrollment files and match by diag yr, age, and enrollment duration in GHC. Ref date = that of the case
HRT use info 2 ways: pharmacy db and for longer duration pre enrollment, via GHC Breast questionnaire (completed by 85%
q Oral hormone use apparently not daily but 25 days a month since sequential described that way shown above) pill types were converted to equivalencies to CEE and MPA for measures of months of use by dose.
q Women were considered premenopausal and excluded if they reported menstrual periods after the ref date.
Hence this study does not consider breast cancer in

Notes4Text

Notes4Marketing

Notes4Graphics

Notes4Permissions

Created

Modified 6/2/2008 5:31:13 PM Unit 1

Word_Doc_Name
