# Supplementary material for: Invasive Breast Cancer Incidence in 2,305,427 Screened Asymptomatic Women: Estimated Long Term Outcomes during Menopause Using a Systematic Review
Source: PLoS One. 2015 Jun 24;10(6):e0128895. doi: 10.1371/journal.pone.0128895 (PMC4479875; doi:10.1371/journal.pone.0128895)
Supplement: S1 Text — (DOCX) [file pone.0128895.s005.docx]

*Appendix D1 providing some more information about these 4009+69 papers with clarification of how it fits in with the initial search strategy.*

This entire project was guided by the scholarly trajectory of the first author, who had been studying and then cataloging the field of reproductive and behavioral endocrinology of aging in women well before the onset of computers. Consequently, this research was carried out in the pre-Cochrane era, during which period she wrote detailed outlines of the methods and results of each study she tracked. She began in 1974 and indexed these studies in an evolving cross-referencing system. The original system was kept on 3x5 cards, as was common before the advent of personal computers. By 1984 she began drafting her outlines on a computer. In the process of compiling and outlining these papers, she included rigorous findings about breast cancer diagnoses in studies that focused on other topics. One such study was on hormone concentrations in elderly women with osteoporosis (See Study 12 [28]). Although these published papers were not necessarily indexed as breast cancer screening studies *by their authors*, they are relevant for the current study on the emergence of breast cancer in asymptomatic women.

In 2008 she transferred 1275 of the outlines that had been prepared on Microsoft Word™ documents into a Filemaker™ system relational database, in preparation for collating the 880 references cited in the 2009 book (see Appendix A, Book 7). The entire set of 4409 references combined the results of that Filemaker™ search with those of the earlier (1974-1984) outlines (on 3x5 cards) and can also be identified from the reference listings in the breast cancer chapters of 5 of the earlier textbooks shown in Appendix A. (See Books 2 through 6).

More recent papers identified by coauthors from their medical society updates were added to these after the Filemaker™ 2008 upload. Dr. Kolter and Burki subsequently selected specific search terms reflecting what had been used in the papers they already had to identify papers we had missed. The search did pick up those few papers that had not been uncovered earlier. Altogether, as shown in Appendix A 4009 + 69 articles were initially searched for relevant studies as referenced in the article (See Fig.1).

Appendix D2, D3 and D4 (see S4. S5 and S6) which offer related elements:

1. A screenshot that lists 276 of the articles (Appendix D2 for step 1 from Filemaker™ card refs copy.pdf)
2. A screenshot showing an individual Filemaker™ electronic “card” (Appendix D3 sample fp5- card ref list sample Chen.pdf)
3. A sample outline from a Microsoft Word™ file (c-Chen W 2006 BrCa ET.doc) (Appendix D4) that was loaded into the Filemaker™ system.
