# Supplementary material for: Invasive Breast Cancer Incidence in 2,305,427 Screened Asymptomatic Women: Estimated Long Term Outcomes during Menopause Using a Systematic Review
Source: PLoS One. 2015 Jun 24;10(6):e0128895. doi: 10.1371/journal.pone.0128895 (PMC4479875; doi:10.1371/journal.pone.0128895)
Supplement: S2 Text — (DOC) [file pone.0128895.s006.doc]

Chen WY, Manson JE, Hankinson SE, Rosner B, Holmes MD, Willett WC, Colditz GA (2006) Unopposed estrogen therapy and the risk of invasive breast cancer. Arch Intern Med 166:1027-1032.

Using the Nurses health Study which enrolled 121,700 RNs 30-55 in 1976, this analysis was limited to postmenopausal women who **had had a hysterectomy**

They defined **Estrogen Therapy (ET) as the use of oral unopposed conjugated equine estrogen therapy.** Those few women who use other types of PMH were analyzed separately and not in this report

The focus on this study is on current use with past users considered separately in a future report planned.

Primary end point was the diagnosis of invasive breast cancer.

Analysis also broken into women who had a recent screening mammogram to confine results to those who had recent ones

RESULTS See tables showing detail

1. Never users were less likely to have history of benign breast disease or to have undergone screening within the past 2 years
   1. And more likely to have a family history of breast cancer
2. Among current users of CEE there was a linear increase in breast cancer risk with increasing duration of ET use (P for trend <.001) although the RR did not become statistically significant until current use exceeded 20 years (RR, 1.42) T2
3. Assn looks stronger for ER+/PR+ cancers but not stat. Signif
4. The ET assn was seen mainly in postmenop women with BMI <25, with RR of 1.77 for >20 yrs use, but >BMI women did not show a sig  RR @ 20 yrs
5. There did not seem to be a strong dose response relationship but power was limited [small proportion of women <.625 or ≥1.25]
6. Consistent with the WHI results, there was a statistically non sig  RR among current ET users for 5 to 9.9 yrs (RR .87 (95% CI .71-1.07.
   1. Same thing found in group ≥60 yrs old

COMMENTS

1. Higher mammography rates are not likely to account for difference since both the regularly screened and not recently screened (more attenuated) showed significant RR for ET use.

WC concludes that conjugated equine estrogens alone do carry an increased risk for breast cancer in long term users but this does not extrapolate to other estrogens

Wednesday, September 27, 2006
