# Supplementary material for: Invasive Breast Cancer Incidence in 2,305,427 Screened Asymptomatic Women: Estimated Long Term Outcomes during Menopause Using a Systematic Review
Source: PLoS One. 2015 Jun 24;10(6):e0128895. doi: 10.1371/journal.pone.0128895 (PMC4479875; doi:10.1371/journal.pone.0128895)
Supplement: S3 Text — (DOCX) [file pone.0128895.s007.docx]

The UK TEDBC [10] study published results of 119,908 mammographic screenings of 39,773 cancer-free women in four age groups. Altogether, 459 women were found to have invasive breast cancer, 1.15% of entire group. A close look at these data reveal the disproportionate impact of a first screening on the cumulative total incidence and highlight how a first screening “finds” breast cancers that may have been present for many years and never recognized until a screening was undertaken. Thereafter only new and clearly much fewer breast cancers will be detected

**Table 3: Breast-cancer detection rates at screening (all cohorts) copied from** Moss SM, for the UK Trial of Early Detection of Breast Cancer Group: 16-year mortality from breast cancer in the UK trial of early detection of breast cancer. *Lancet* 1999; 353:1909-14.

| \| **Age at Screening** \| **Number Screened** \| **Invasive Cancer (rate per 1000)** \| **In-situ cancer (rate per 1000)** \| \| --- \| --- \| --- \| --- \| \| **First Screening** \|  \|  \|  \| \| 45-49 years \| 15868 \| 41 (2**⋅**6) \| 21 (1**⋅**3) \| \| 50-54 years \| 8543 \| 37 (4**⋅**3) \| 6 (0**⋅**7) \| \| 55-59 years \| 8442 \| 36 (4**⋅**3) \| 7 (0**⋅**8) \| \| 60-64 years \| 6636 \| 45 (6**⋅**8) \| 12 (1**⋅**8) \| \| > 65 years \| 284 \| 5 (17.6) \| 0 \| \| Total \| 39773 \| 164 (4**⋅**1) \| 46 (1**⋅**2) \| \|  \|  \|  \|  \| \| **Mammographic Rescreening** \|  \|  \|  \| \| 45-49 years \| 11390 \| 23 (2**⋅**0) \| 6 (0**⋅**5) \| \| 50-54 years \| 20638 \| 42 (2**⋅**0) \| 8 (0**⋅**4) \| \| 55-59 years \| 19354 \| 64 (3**⋅**3) \| 17 (0**⋅**9) \| \| 60-64 years \| 17859 \| 49 (2**⋅**7) \| 10 (0**⋅**6) \| \| > 65 years \| 10894 \| 41 (3**⋅**8) \| 8 (0**⋅**7) \| \| Total \| 80135 \| 219 (2**⋅**7) \| 49 (0**⋅**6) \| \|  \|  \|  \|  \| |
| --- | --- | --- | --- | --- | --- | --- | --- | --- | --- | --- | --- | --- | --- | --- | --- | --- | --- | --- | --- | --- | --- | --- | --- | --- | --- | --- | --- | --- | --- | --- | --- | --- | --- | --- | --- | --- | --- | --- | --- | --- | --- | --- | --- | --- | --- | --- | --- | --- | --- | --- | --- | --- | --- | --- | --- | --- | --- | --- | --- | --- | --- | --- | --- | --- | --- | --- | --- | --- |
|  |
