# Supplementary material for: Invasive Breast Cancer Incidence in 2,305,427 Screened Asymptomatic Women: Estimated Long Term Outcomes during Menopause Using a Systematic Review
Source: PLoS One. 2015 Jun 24;10(6):e0128895. doi: 10.1371/journal.pone.0128895 (PMC4479875; doi:10.1371/journal.pone.0128895)
Supplement: S4 Text — (DOCX) [file pone.0128895.s008.docx]

**Weighing your risk**

Although epidemiologists have used the term for more than 30 years [^per Dr. Janet Dahling^] a relatively new term in the gynecology journals, **attributable risk,** has appeared. Attributable risk refers to the incidence, (in this case, breast cancer) in women with a behavior (in this case, hormone therapy) minus the incidence without the behavior. To calculate the attributable risk of hormone therapy on cancer, first one would have to know the incidence of cancer. For the WHI data, that incidence was 30 cases per 10,000 per year for “no-hormone” users at baseline.^(r391)^ Since there were 8 extra cases in the hormone users, 38 per 10,000, the attributable risk for those who used Prempro^®^ was 8 per 10,000 women per year.

Another way of describing the **attributable factor** is: the proportion of the disease due to a particular factor. Using data from the Million Women Study, this was calculated to be 3.27% additional risk with combination therapy after 15 years of use. ^(r154)^ One should note it is not 3.27% of the women who will get this cancer, but rather 3.27% additional of the 2% that will get cancer.

3.27% X 2% = .06%

In English this is 6 one-hundredths of one percent. I.e. extremely tiny numbers. Table 12-2^(r23)^, compares some risk factors now known to increase the risk of breast cancer showing their relative influence. Excess alcohol, lack of exercise and excessive weight gain have much greater influence on a woman’s potential to get breast cancer.

**Table 12-2: Behaviors that Influence Breast Cancer –Attributable Risks**

| **Risk Factor over 20 Years**  **For Women 50-70 Years Old** | **Breast Cancer Cases** | **Extra Cases** |
| --- | --- | --- |
| Never Used HRT | 45/10,000 |  |
| > 5 yrs HRT | 47/10,000 | 2/10,000 |
| > 10 yrs HRT | 51/10,000 | 6/10,000 |
| >15 yrs HRT | 57/10,000 | 12/10,000 |
| Menopause after age 60 | 59/10,000 | 14/10,000 |
| Alcohol (2 drinks/day) | 72/10,000 | 27/10,000 |
| No daily exercise | 72/10,000 | 27/10,000 |
| Weight gain (>20 kg) | 90/10,000 | 45/10,000 |

**Different HRT regimens have different attributable risks**

I don’t think you can take “hormone use” at face value, because they vary. I think we should differentiate regimens in discussing attributable risk and breast cancer. Here’s why.

Beginning in 2002, a series of scientific analyses were conducted to study which hormone regimens are most risky.

**What**: The National Institutes of Health Contraceptive And Reproductive Experience (CARE) study, one of the few studies to examine breast cancer risk in relation to specific hormone regimens. This was a multicentered population-based case-control study conducted in 5 United States metropolitan areas (Atlanta, Detroit, Los Angeles, Seattle and Philadelphia) from 1994 – 1998.

**Who**: 3,823 cases of breast cancer compared to 1953 local contemporaries without cancer.^(r832)^ It collected detailed information on lifetime use of hormones from a large number of women residing in 5 geographical areas of the U.S..

**What they found**: The association between risk of breast cancer and hormone replacement therapies varies by regimen.

The United States multi-centered study reported that *only continuous combined HRT for 5 years or more (the Prempro*^®^ *type) increased the risk, which worsens with increasing duration of use.* *The risk appeared to evaporate with discontinuation of the regimen*. Former users had no elevation in risk of breast cancer. Nor did the women who used sequential progestins opposed to their estrogens. And only current users of continuous combined regimens were at risk. Apparently, this risk was confined to the rarer lobular type of breast cancer.^(r181-r182)^

The continuous combined hormone therapies outcome in CARE observational studies ^(r181-r182-r561-r736)^ were roughly similar to the outcomes of the Women’s Health Initiative and observational studies which found an increased risk of breast cancer detection in Prempro^®^ users.

**More proof**: Confirming data on the deleterious effects of continuous combined regimens with the first invasive breast cancer were reported in another study of older women, 65-79, in the Seattle metropolitan area.

**What they found**: Unopposed estrogen replacement therapy did not increase the risk of breast cancer even up to 25 years or more of use. *But continuous combined therapy (with MPA as the predominant progestin) of more than five years did increase the risk of both ductal and lobular breast cancer.*^(r449)^ Further studies determined that prior oral contraceptive use appeared to be protective for postmenopausal hormone therapy use in reducing the likelihood of breast cancer.^(r561)^

**Another attributable risk: Absence of Sunlight UVB-radiation**

Sunlight, the principal source of Vitamin D (see CH 8) has long been recognized to be a risk factor for skin cancer. As a consequence many dermatologists and media urge women to avoid the sun, to block it with sun screens. One investigator noticed that there were large geographic variances in the United States in mortality rates for many cancers. In fact, except for skin cancers, he noted that breast, colon, ovary and prostate, non-Hodgkin lymphoma, bladder, esophageal, kidney, lung, pancreatic, rectal, stomach and uterine cancers were regionally variable in the United States. The rates were approximately twice as high in the northeast versus the southwest.^(r291)^ He concluded that many lives could be extended with solar exposure or Vitamin D3 supplementation. *I agree.*

**What you should know:** Additionally, the scientists reviewed breast cancer mortality rates from 1989-1996, along with dietary supplement data and latitude (which is an index of solar UVB) from 35 countries.^(r290)^ They calculated that 80% of the variance of breast cancer mortality rates was explained in this order:

• fraction of calories derived from animal products (more risk),

• fraction of calories derived from vegetable products (reduction in risk)

• followed by solar UVB, (reduction in risk)

• alcohol use (increased risk)

• fish intake (reduction in risk).

37 Cutler WB. *Weighing your Risk, pages 204-207* in [Hormones and Your Health: The Smart Woman's Guide to Hormonal and Alternative Therapies for Menopause](http://www.athenainstitute.com/hormonesandyourhealth.html), John Wiley, NY, 2009.
